# Adaptation, Implementation Plan, and Evaluation of an Online Tobacco Cessation Training Program for Health Care Professionals in Three Spanish-Speaking Latin American Countries: Protocol of the Fruitful Study

**DOI:** 10.2196/resprot.6487

**Published:** 2017-01-27

**Authors:** Cristina Martínez, Assumpta Company, Olga Guillen, Mercè Margalef, Martha Alicia Arrien, Claudia Sánchez, Paula Cáceres de León, Esteve Fernández, Group of Hospital Coordinators in the Fruitful Project

**Affiliations:** ^1^ Institut Català d’Oncologia-ICO Tobacco Control Unit, Cancer Control and Prevention Programme L'Hospitalet del Llobregat Spain; ^2^ Cancer Control and Prevention Group Institut d’Investigació Biomèdica de Bellvitge-IDIBELL L’Hospitalet de Llobregat Spain; ^3^ Universitat Internacional de Catalunya Medicine and Health Sciences School Sant Cugat del Vallés Spain; ^4^ Institut Català d’Oncologia-ICO Training Unit L’Hospitalet de Llobregat Spain; ^5^ Institut Català d’Oncologia-ICO Tobacco Control Unit, Cancer Control and Prevention Programme L’Hospitalet de Llobregat Spain; ^6^ Management Department Instituto Oncologico del Oriente Boliviano de Santa Cruz de la Sierra Santa Cruz de la Sierra Bolivia; ^7^ Public Health Department Ministerio de Salud y Pública y Bienestar Social Asuncion Paraguay; ^8^ Radiation Oncology Department Instituto de Cancerología y Hospital Dr. Bernardo Guatemala Guatemala; ^9^ School of Medicine Department of Clinical Sciences Universitat de Barcelona L'Hospitalet del Llobregat Spain

**Keywords:** tobacco cessation, online, training, low- and middle-income countries, policies

## Abstract

**Background:**

Tobacco cessation training programs to treat tobacco dependence have measureable effects on patients’ smoking. Tobacco consumption in low- and middle-income countries (LMICs) is high and slowly decreasing, but these countries usually lack measures to face the epidemic, including tobacco cessation training programs for health professionals and organizations. Based on a previous online smoking cessation training program for hospital workers in Spain, the Fruitful Study aims to increase smoking cessation knowledge, attitudes, self-confidence, and performance interventions among health care professionals of three Spanish-speaking low- and middle-income Latin American and Caribbean (LAC) countries.

**Objective:**

The purpose of this paper is to describe the methodology and evaluation strategy of the Fruitful Study intended to adapt, implement, and test the effectiveness of an online, evidence-based tobacco cessation training program addressed to health professionals from Bolivia, Guatemala, and Paraguay.

**Methods:**

This study will use a mixed-methods design with a pre-post evaluation (quantitative approach) and in-depth interviews and focus groups (qualitative approach). The main outcomes will be (1) participants’ attitudes, knowledge, and behaviors before and after the training; and (2) the level of implementation of tobacco control policies within the hospitals before and after the training.

**Results:**

To date, adaptation of the materials, study enrollment, and training activities have been completed. During the adaptation, the main mismatches were language background and content adaptation. Several aids were developed to enable students’ training enrollment, including access to computers, support from technicians, and reminders to correctly complete the course. Follow-up data collection is in progress. We have enrolled 281 hospital workers. Results are expected at the beginning of 2017 and will be reported in two follow-up papers: one about the formative evaluation and the other about the summative evaluation.

**Conclusions:**

There is a need to learn more about the cultural and content elements that should be modified when an online tobacco cessation training program is adapted to new contexts. Special attention should be given to the personal and material resources that could make the implementation possible. Results from the Fruitful Study may offer a new approach to adapting programs to LMICs in order to offer education solutions with the use of emerging and growing communication technologies.

**ClinicalTrial:**

Clinicaltrials.gov NCT02718872; https://clinicaltrials.gov/ct2/show/NCT02718872 (Archived by WebCite at http://www.webcitation.org/6mjihsgE2)

## Introduction

### Background

Tobacco use remains a global public health concern; annually it causes 6 million preventable deaths [1]. The tobacco consumption epidemic is shifting to low- and medium-income countries (LMICs) such as some countries in the Latin American and Caribbean (LAC) region [2]. Currently, more than 120 million smokers live in these countries [3]; half of them will develop a tobacco-related disease and consequently will require medical care.

In the LAC region, smoking rates vary by country, sex, and socio-economic status [4,5]. In some low-income countries, such as Bolivia, Guatemala, and Paraguay, smoking rates are 10 percentage points higher than the rest of the LAC countries [6]. Thus, among men, smoking prevalence ranges from 42% (in Bolivia) to 22.9% (in Paraguay) [7]. Among women, the prevalence is lower, but it is rapidly increasing, confirming the alarming feminization of the epidemic in LAC countries [7]. In the overall region, smoking-related mortality accounts for 16% of total mortality [7], and according to the World Health Organization (WHO), smoking-related deaths will increase by 700% by 2030 [8]. The report points out that the epidemic can be curbed by implementing comprehensive tobacco control measures embraced by the WHO Framework Convention on Tobacco Control (WHO-FCTC) [9]. These measures have demonstrated the reduction of tobacco use and the increase of awareness of its hazards [9]. Guatemala, Paraguay, and Bolivia signed the WHO-FCTC early on, and have implemented some tobacco control measures, including smoke-free legislation (according to the WHO-FCTC Article 8) in workplaces and public places, including health care services. However, smoking cessation services (Article 14) have not received the same recognition and attention [5]. As a consequence, smoking cessation interventions are not well spread among health care services within these countries. Smokers are frequent users of health care services, and their contact with the health system might be an adequate “teachable moment” for quitting [10]. Indeed, according to studies conducted in the United States between 60% and 70% of patients make an attempt to quit while they are hospitalized [11]. However, in spite of these favorable conditions, evidence-based cessation programs are hardly available in LAC countries [12]. The most common barriers to incorporating tobacco cessation interventions into hospitals involve lack of training, expertise, and time. In addition, organizational and financial constraints threaten the suitability of smoking cessation interventions [6].

In LAC countries smoking consumption among health care professionals is still similar to that of the general population [13,14], and most doctors and nurses acknowledge that they have not received formal training in smoking cessation during their undergraduate or graduate training [14]. Generally, they state little confidence in its effectiveness to help their patients to stop smoking. Training facilitates having a more positive attitude on smoking cessation [15] and helps to increase the performance of smoking cessation interventions [16]. Given that about 70% of smokers visit health services over the course of a year, the lost opportunities to intervene remain significant [3]. Frequently, providers believe that smoking cessation is extremely important, but also extremely difficult due to a serious communication gap between doctors and patients that jeopardizes the opportunities to support smokers to quit [17].

Implementation research recommends addressing organizational constraints in order to overcome executive barriers when the cessation messages are delivered such as lack of time, support, and resources [18,19]. Training programs obtain higher impact and sustainability when they are fostered by organizations that allocate time, promote key champions, and provide implementation materials and resources [20,21]. At the organizational level, the chief executive should endorse the initiative and disseminate this endorsement through senior management meetings and routine communication mechanisms. In addition, an implementation committee led by a champion and members of the executive board should establish a feasible execution plan. Following this strategy, tobacco cessation training programs would better sway professional norms and promote the implementation of smoking cessation services.

In the literature, there are several conceptual training models that exhibit significant heterogeneity [22]. For example, online courses allow for distance-learning, are cost-efficient, and provide modes to teach and reinforce counseling skills that often can be difficult to convey in traditional classroom settings [23,24]. Moreover, online training has clear potential to meet large-scale training dissemination needs. Compared with classroom instruction, online training offers greater learner accessibility, increased convenience, and greater scalability [22]. Previous online tobacco cessation training courses have demonstrated an increase in the health provider's skills to counsel patients on tobacco cessation [16,25,26]. Although there are several distant learning tobacco cessation training programs the majority have been developed and evaluated in English-speaking countries. A recent review on online tobacco dependence treatment training programs (all in English) found that 17 out of the 24 courses evaluated failed to meet minimal quality standards. The authors suggested improving instructional design elements, such as teaching effectiveness, learning strategies, instructor’s role and assessment, and evaluation [27].

Using previously established programs can save time and money while increasing the likelihood of achieving successful outcomes [27]. In addition, research training initiatives have been suggested to increase capacity-building efforts, in particular within developing countries [22]. However, the evidence of distant learning training from high-income countries (HICs) may not be directly applicable to LMICs and may need cultural and content adaptations. Research is needed to develop and understand how best to implement effective smoking cessation strategies in LMICs, especially in resource-poor environments where access to health care providers are limited.

### Conceptual Framework

Adaptation implies not only to the replication or translation of a program but to the process of adjusting a program to reduce mismatches between its characteristics and the new context in which it is to be implemented. Adapting a tobacco cessation education program should take into account the treatment regulation of each country, the existing guidance and proceedings, and the dynamics of the health organization [3]. Card et al [28] propose science-based pragmatic steps to adapt an existing program to new contexts based on the following steps: (1) select a suitable, effective program; (2) gather the original program materials; (3) develop a program model; (4) identify the program score components and best-practice characteristics; (5) identify and categorize mismatches between the original program model or materials and the new context; (6) adapt the original program model, if warranted; and (7) adapt the original program materials. To fill the gap of the lack of tobacco cessation training programs in Spanish-speaking LAC countries, we designed the “Fruitful study.”

### Prior Work

We selected the ongoing Brief Intervention for Smoking Cessation Training Program from the Catalan Institute of Oncology (ICO) as the baseline program and created a partnership between ICO and three hospitals from Bolivia, Paraguay, and Guatemala to first adapt and later disseminate the course. We intend to increase the capacity-building of the implicated organizations that will ultimately initiate a cascade of change within their countries [29]. Our training is intended to be a vehicle for the systematic dissemination of the clinical practice guidelines for treating tobacco dependence in hospitalized and ambulatory smoker patients.

The purpose of this paper is to describe the methodology and evaluation strategy of the Fruitful Study research protocol intended to adapt, implement, and test the effectiveness of an online, evidence-based tobacco cessation training program addressed to health professionals from Bolivia, Guatemala, and Paraguay.

### Research Objectives

The primary research goal of the Fruitful Study is to evaluate the impact of an online, evidence-based tobacco cessation training program aimed to increase smoking cessation knowledge, attitudes, self-confidence, and performance interventions among health care professionals of three Spanish-speaking low- and middle-income LAC countries (Bolivia, Guatemala, and Paraguay).

### Hypothesis

Our primary hypothesis is the participants in the online tobacco cessation training program will increase their tobacco-related knowledge, attitudes, and behaviors by the 6-month follow-up, as compared to baseline conditions prior to the training. Our secondary hypothesis is that participating hospitals will exhibit greater tobacco control progression, commitment, and implementation of tobacco cessation services.

## Methods

### Study Design

The Fruitful Study is a 2-year mixed-method (qualitative and quantitative) study conducted in one hospital in each of the three participant countries (Bolivia, Guatemala, and Paraguay).

For the quantitative approach, a pre-post design will be used to (1) assess participants’ attitudes, knowledge, and behaviors before and after the training using a questionnaire (Tool 1); and (2) measure the level of implementation of tobacco control policies within the participant hospitals before and after the training using the Self-Audit Questionnaire (SAQ) (Tool 2). For the qualitative approach, focus groups and in-depth interviews of key persons will be used. With this methodology we aim to (1) explore the experience of adapting the training program in each of the participant hospitals; (2) ascertain participants’ experience undertaking the training program; and (3) understand the opportunities and barriers of undertaking online smoking cessation training programs in the participant countries. With the results of the focus groups, the research team aims to identify any difficulties in accessing and completing the course and to make the necessary improvements.

### Participants

The two main participant units from each organization are students and the hospitals themselves. The participant hospitals were selected because they had previously collaborated with the Training Unit of the ICO in training their health professionals in either electronic learning (e-learning) or in-person courses. The selected hospitals and their characteristics are shown in Table 1.

**Table 1:** Characteristics of the selected hospitals.

Hospital	Country	Type of hospital	Workers, n	Beds, n
Instituto Oncológico del Oriente Boliviano de Santa Cruz de la Sierra	Bolivia	Public, urban oncology hospital	359	79
Instituto de Cancerología y Hospital Dr Bernardo del Valle	Guatemala	University, public, urban oncology hospital	300	108
Instituto Nacional de Enfermedades Respiratorias y del Ambiente (INERAM)	Paraguay	University, public, respiratory hospital	746	151

Student participants were all health professionals and paraprofessional staff from the three participant hospitals. All health providers in the selected hospitals, including nurses, doctors, and other health professionals were invited to enroll in the training. Each local coordinator recruited participants from a variety of units and departments over a 6-month period through informative sessions, leaflets, and posters (designed to inform about the training program).

### Procedure and Timeline

#### Selecting a Suitable and Effective Training Program

The selected original training program was chosen because it was shaped using evidence-based guides, was originally designed for Spanish hospitals (culturally and organizationally similar to LAC hospitals), and is addressed to hospital workers. Furthermore, offering the course through an online platform has clear potential to reduce cost and increase training coverage within participant hospitals in less time.

**Figure 1:**
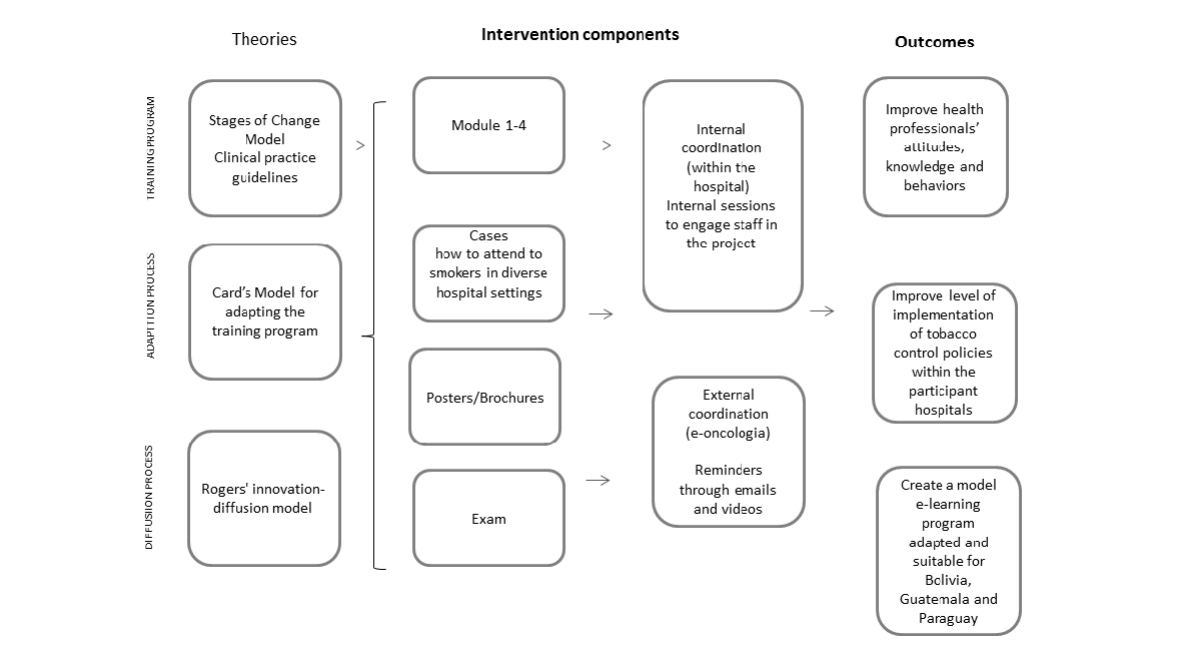
Conceptual framework.

**Figure 2:**
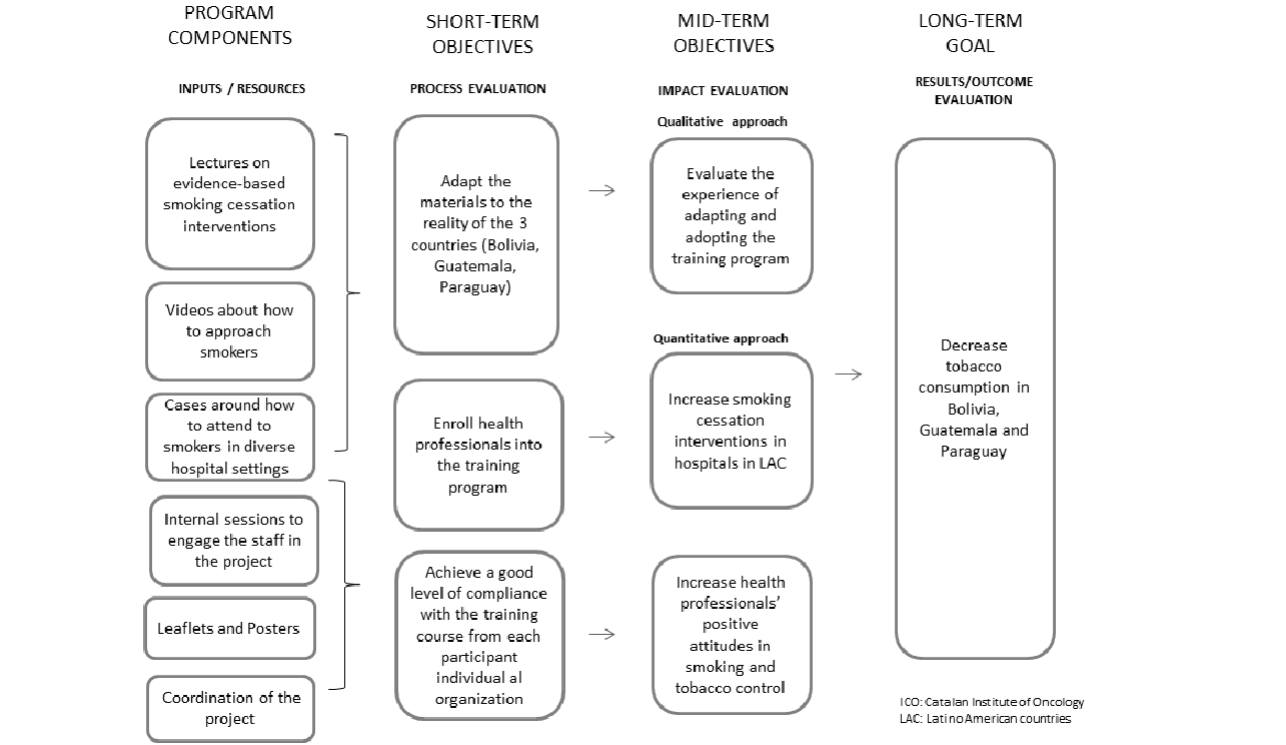
Program model.

The original course was developed in the online platform e-oncologia based on the in-person courses offered during the last 10 years by the Tobacco Control Unit of the ICO. The theoretical framework underpinning the training program is the Stages of Change Model [30], and the curriculum was developed with the content of numerous meta-analysis and clinical practice guidelines [16,31-34] (Figure 1). We created a fully referenced, online curriculum, with feedback from an expert advisory group that oriented the instructional design to ensure the course content was palatable for an online format and aligned with the learning objectives. The goals and program components (strategies, activities, services, etc) are depicted in Figure 2. The final curriculum content of the “Brief Intervention for Smoking Cessation Training Program” is composed of 4 modules (Textbox 1).

**The 4 modules of the Brief Intervention for Smoking Cessation Training Program**

Modules

- Module 1 describes the tobacco epidemic, tobacco-related morbidity and mortality, second hand smoke, and measures included in the MPOWER strategy to tackle smoking.
- Module 2 provides orientation on how to assess the smoker, how to assess tobacco dependence and willingness to quit, and evaluate smoker self-efficacy, previous quit attempts, previous relapses, and so on.
- Module 3 introduces the efficacy of the different levels of attention and treatment orientations (eg, cognitive behavioral, psychodynamic, medication management) and presents the clinical settings where the intervention is possible (eg, inpatient, outpatient, ambulatory treatments). It explains in detail the 5As intervention model.
- Module 4 explains in detail the different tobacco cessation treatments available (nicotine replacement, bupropion, varenicline, and other treatments). It also provides orientation about the follow-up, strategies to improve the adherence of the treatment, how to identify withdrawal symptoms, how to deal with relapses, and so on.

This online training program includes (1) slides; (2) review exercises; (3) cases of 4 patients differing in demographics, diagnostic, stages of change, setting; and (4) problem solving exercises. The training provides several materials including slides, online tutorials with an expert tutor, recommended readings, patient cessation brochures, a therapeutic pocket guideline, and an organizational recommendation model to facilitate the implementation of tobacco cessation services in the hospital setting.

The original online course was firstly tested by 10 voluntary participants in Catalonia (Spain). Evaluation of the tobacco cessation program was thereafter tested in the ICO by 150 health professionals. This pilot test proved the acceptability of the training model, the adequacy of its contents, and obtained a high level of satisfaction in the trainees. This training initiative has also been shown to increase the level of implementation of tobacco control in Catalan hospitals (according to the SAQ) [35] and the engagement of health professionals [36,37], thus making them part of the solution. This course has been accredited by the Council of Oncology in Europe (ACOE) in support of Continuing Medical Education for physicians. Since its launch in November 2012 to June 2014 more than 1000 Catalan health professionals have taken this course.

Based on literature regarding attrition among online learners [38], and our own experience delivering online training to Catalan health workers, we anticipate that roughly 50% of participants will complete all components of the proposed activities.

#### Adaptation of the Material

The adaptation has been done with local partners and other stakeholders. First, a group of professionals with extensive experience in tobacco control in their country were selected for identifying and categorizing mismatches between the original materials and the new context. Experts expressed that the goal and objectives were aligned with the original program model (Figure 2). Since they approved the interface and the images employed as culturally acceptable, the same layout was used. However, we detected some mismatches (Textbox 2) that were modified in a version that was pilot tested by 8 to 10 volunteers in each country.

**Identified mismatches that were later modified and pilot tested**

Mismatches

- Language background and literacy level in some of the terms used:
  The course was in Spanish from Spain, and the Spanish spoken in Bolivia, Guatemala, and Paraguay differs somewhat with respect to vocabulary, some expressions, and in some cases even in grammar structures.
- Description of the epidemiology smoking in Model 1:
  The original version included the situation in Spain, whereas the adapted version included the most updated data in Bolivia, Guatemala, and Paraguay.
- Tobacco cessation pharmacological treatment in Module 4 (nicotine replacement therapy, bupropion, varenicline) and settings where tobacco prevention and cessation services are performed in Spain (primary care, hospitals, quitlines, etc) were adapted to the current resources in each country.
- In case studies, the clinical simulations demonstrate the cultural characteristics of each country.
- Questions and answers of the assessment and evaluation were also changed according to the adapted contents.

Several aids were necessary in order to enroll students. Thus, local investigators suggested a double registration method through the online platform consisting of an independent method (first one) by which students generated their own credentials and a second one assisted by a computer technician who created the login credentials and helped them in the usage of platform. The second method was necessary since many students had little or no experience in pursuing online education.

#### Implementation Plan

**Figure 3:**
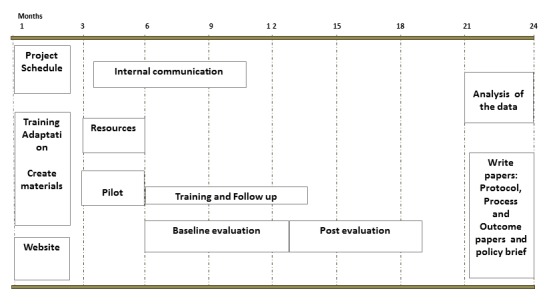
Event planning timeline.

This study is expected to last 24 months (from November 2014 to November 2016). Information on the various activities is depicted in the event planning timeline (Figure 3).

This paper was written during the 18th month of the study when the post-evaluation data was being gathered. During the training (months 6 to 13), each participant had 1 month to fill in the baseline evaluation, complete the online training, and take the exam. Participants were monitored by local coordinators that acted as champions and encouraged participants to enroll and complete the course. Coordinators set monthly meetings with personnel from the different units and services to increase the number of participants and support those enrolled to complete the course. In addition, during the implementation, coordinators from each hospital offered assistance to students with little or no computer skills. Technical support was mainly used by less educated health professionals (assistants and support health professionals) and older workers who were less familiar with the use of computers. They offered their assistance in logging into the online platform, filling out the questionnaires, completing the evaluation, as well as other technical support-related issues. Participants’ progress was monitored in real-time on the Web platform. The project coordinator at the ICO sent biweekly reports of the participants' progress to coordinators, and if necessary personal emails and videos that motivated the students to finish the course and complete the evaluations. Participants accessed the course using their electronic devices (mobile phones, tablets or computers) or the ones provided by their hospitals during and after working shifts. The hospitals from Guatemala and Paraguay made a library available with computers to facilitate students’ enrolment before or after their working hours. The hospital from Bolivia provided a computer room in which students had access to the course and the support of a technician during specific hours of the day. Therefore, the participating hospitals facilitated computer and Internet access at the workplace.

#### Evaluation Design

To design the evaluation we applied the conceptual frame for continuing medical education (CME) conceived by Moore et al [39] that fosters meaningful approaches to address the issues of health professional competence and performance. From the 7-level outcome framework, we employed those from level 1 to level 5 as follows: (1) participation (measured by coverage fulfillment of the course); (2) satisfaction (measured through a satisfaction questionnaire); (3) A-learning and B-learning procedural and declarative knowledge (measured using a pre-post test of knowledge); (4) competence (measured using a pre-post self-report on competence); and (5) performance (measured using a pre-post self-evaluation report of performance). In the evaluation formative and summative assessments will be used.

##### Formative Evaluation

The formative evaluation will assess the adoption, implementation, and maintenance of the training program. We will measure program coverage, completion rates, fidelity with the training program, usage of the materials, and the satisfaction with the training. We will use qualitative (focus groups and interviews) and quantitative methods to gather this information. The indicators linked to the performance of the program are (2) number of participants; (2) characteristics of the participants (profession, units, sex, age); (3) number of hours dedicated to the training program; (4) program performance and fidelity to the curriculum plan (whether the students completed all the modules, the exercises as planned); (5) service utilization or dosage use of the training (time applied for undertaking the course, number of downloads of the materials, etc); and (6) opinions, experience, perceptions, and satisfaction with the training course.

##### Summative Evaluation

We will investigate the short and intermediate outcomes by measuring the impact of the smoking cessation training program within the participant hospitals using quantitative methods (pre-post design). This will include the level of implementation of tobacco control policies within the participant hospitals before and after the training by using the SAQ (Q1) and participants’ attitudes, knowledge, and behaviors before and after the training using a questionnaire (Q2).

### Instruments

#### Tool 1

To assess differences in tobacco control polices implemented within the hospitals we will use the European Network of Smoke free Hospitals (ENSH) SAQ [35]. This tool was developed for the ENSH-Global Network for Tobacco Healthcare Services. The questionnaire is composed of the following 10 policy standards: (1) commitment (6 items); (2) communication (4 items); (3) education and training (4 items); (4) identification and cessation support (8 items); (5) tobacco control (5 items); (6) environment (6 items); (7) healthy workplace (5 items); (8) health promotion (1 item); (9) compliance monitoring (2 item); and (10) and policy implementation (1 item). Each item is scored 1 (not implemented), 2 (less than half of the aspects are implemented), 3 (more than half are implemented), or 4 (fully implemented). The maximum score of the ENSH SAQ is 168 points, calculated as the sum of the 10 standards [35]. At baseline, the SAQ provides information on the tobacco control policies undertaken within the organization. Once it is used to monitor the project, the instrument detects the fulfilled standards and the areas for improvement.

#### Tool 2

Trainers’ attitudes, knowledge, and behaviors will be assessed using a questionnaire composed of 63-items. The website-delivered questionnaire is emailed to the participants at baseline and 3 months after finishing the training. The questionnaire, designed by Sheffer et al [40], takes 30 to 40 minutes to complete. The questionnaire gathers information about the provider's sex, tobacco use history, previous tobacco cessation education, level of pro-activity addressing tobacco use, and perceived success in helping patients stop using tobacco [40]. Perceived knowledge and attitudes about treatment of tobacco use will be determined by assessing the levels of (1) motivation; (2) knowledge about tobacco cessation; (3) self-efficacy; (4) importance of providing tobacco use interventions; (5) effectiveness of interventions; (6) importance of barriers; (7) readiness; and (8) level of tobacco cessation intervention provided assessed by the 5A’s model, an evidence-based framework that helps health professionals to structure smoking cessation interventions by identifying all smokers and offering support to help them quit. The 5A's model consists of 5 components: ask, advice, assess, assist, and arrange follow-up. All items are assessed on a discrete scale from 0 (none or not at all) to 10 (the most possible). The pre-test will be administered immediately prior to the training. The post-training assessment is composed of a 37-item questionnaire assessing providers’ knowledge, attitudes, and behaviors, as assessed in the pre-test.

#### Focus Groups

Each participant country will carry out at least 5 focus groups with hospital workers enrolled and not enrolled in the course. Participants will be volunteers. The focus groups will consist of homogenous working categories to avoid possible reticence of some participants to openly talk in the presence of their supervisors. In each country focus groups will be conducted by external local qualitative researchers that will follow the same protocol.

### Data Analysis

Descriptive statistics will be used for the quantitative indicators. The quantitative variables will be summarized using means and other central tendency measurements, whereas the qualitative data will be summarized by computing their frequencies and percentages. The qualitative indicators gathered by the qualitative methods (focus groups and interviews) will be summarized using the classical content analysis approach (creating codes and chunks of information and the researcher complements the codes with description of this code). Analysis of the data will be validated by informants to increase the reliability of the data [41].

Usual statistics will be used to describe the sample and non-parametric tests will be used for pre-post comparisons for tobacco control policies (measured by Tool 1) and the trainees’ knowledge, attitudes, and behaviors (measured by the Tool 2). In addition, a validity and reliability test of both instruments is planned.

### Sample Size

Based on literature regarding gains of training [27,40], we anticipate an increase of 40% in health professionals’ level of knowledge, attitudes, and perception in tobacco cessation from baseline to 6 months. Given this estimate, we will need a minimum of 43 participants per hospital, (total 129, alpha=.05, beta=.1, and 15% dropouts). However, we expect at least an overall enrollment of 300 professionals (100 per hospital).

## Results

We have enrolled 281 hospital workers (105 from Bolivia, 88 from Guatemala, and 88 from Paraguay). The overall average completion rate is 66.2% (186/281). At the time of submission of this paper, data collection for the post-evaluation was underway. Evaluations will finish in November 2016 and results are expected by the beginning of 2017. Results of this study will be reported in two follow-up papers: one about the formative evaluation and the other about the summative evaluation.

## Discussion

### Principal Findings

We will consider a good level of coverage of the training program if at least 50% of the enrolled health professionals in each hospital conclude the training program. We will measure the engagement of the training program with the focus groups and key informant interviews. We anticipate that hospitals will increase their tobacco control policies by 20% to 30% according to the SAQ. In addition, we anticipate that health professionals’ level of knowledge, attitudes, and perception in tobacco cessation will increase to 30% to 40%.

### Limitations and Strengths

Health professionals and paraprofessional staff from the three countries will be invited to voluntarily participate in the project. We will be able to determine whether the recruitment process used by each coordinator affects both the number of participants recruited and the commitment to start and finish on time. We hypothesize that as they agree to join, they will be highly motivated to pursue the course and we expect many of them to succeed on their own. However, participants may have varying levels of computer skills and Internet usage that could affect their course enrollment and progression. Online training programs also require devices for their use (ie, computers, tablets or mobile phones with app capabilities) and a high speed Internet connection. Although Internet services and technological devices are rapidly growing in low-income countries [42] it may not be the same as in HICs. Nevertheless, the course could be followed by these platforms facilitating the connectivity among users (tablets, mobile phones) being able to complete the training program at home or in their working hospitals. We foresee that many of the students will be using mobile phones as their primary access to the course, because mobile phones are more accessible in these countries than computers [42]. Furthermore, the participants could have different knowledge in smoking cessation interventions within participant hospitals and among the different countries. This could make comparisons among participant countries very interesting. The fact that we included baseline and post-intervention evaluations will permit the evaluation of these differences.

### Comparison With Prior Work

According to a paper by Ng et al [43] tobacco use in these three countries is decreasing at a slower pace than in developed countries. Furthermore, tobacco cessation services have been poorly implemented in these countries [5] mainly because of the lack of knowledge and skills of health providers, difficulty to ground tobacco cessation interventions in health care organizations, and lack of working groups and leaders on this topic [6]. Online education in tobacco cessation might be the solution to provide evidence-based treatment for tobacco dependence in these countries because it is cost efficient and can reach remote locations. Most of the existing smoking cessation training programs have been designed in HICs and are available only in English [27]. Nevertheless, the process of spreading new learning approaches requires cultural and content adaptations that require reviewing whether the material and examples are applicable to the new target group [44]. To our knowledge this is the first study that tests the feasibility and effectiveness of implementing an online smoking cessation training program addressed to health care providers in Bolivia, Guatemala, and Paraguay. We thus consider this study approach innovative because (1) it adapts a straightforward smoking cessation training program through an online platform addressed to all hospital workforce levels (from doctors, nurses, and assistance personnel) of three LMICs in LACs; (2) it applies an organizational model in LACs to promote smoking cessation training within the organization; and (3) it evaluates its impact using quantitative and qualitative methods from the individual level (by measuring students’ knowledge, attitude, and behavior gains before and after the training) to an organizational level by measuring whether the organization has increased tobacco control policies before and after the training. Moreover, facilitators and barriers of implementation will be evaluated by using focus groups and in-depth interviewers to key people involved in the project.

It is for these reasons that the methods and results of Fruitful Study may offer a new approach to adapt and implement programs to LMICs countries in order to offer education solutions through the use of emerging and growing communication technologies.

### Conclusions

It is critical to develop distant learning programs to train health workers in tobacco cessation in LMIC where opportunities for continuing education are scant and sometimes not well adapted to the reality of their contexts. This study will show whether it is possible to adapt and implement an online course in developing countries. In addition, we will determine how best to implement effective distant-learning strategies in organizations belonging to LMIC, especially to detect what resources and aids are required to make the training possible in all levels of the workforce. Finally, we will measure whether the training program will increase knowledge, attitudes, and perception in tobacco cessation among participants and will produce changes in tobacco control policies at the organizational level.

## Abbreviations

ENSH: European Network of Smoke free Hospitals
ICO: Catalan Institute of Oncology
HIC: high-income country
LAC: Latin American and Caribbean
LMIC: low- and middle-income country
SAQ: Self-Audit Questionnaire
WHO: World Health Organization
WHO-FCTC: WHO Framework Convention on Tobacco Control
